# Visitor Engagement, Relationship Quality, and Environmentally Responsible Behavior

**DOI:** 10.3390/ijerph17041151

**Published:** 2020-02-12

**Authors:** Xiaoli Zhou, Chengcai Tang, Xingyang Lv, Bo Xing

**Affiliations:** 1 Department of History-Culture & Tourism Management, Changzhi University, Changzhi 046000, China; 15234563932@163.com; 2 School of Tourism Sciences, Beijing International Studies University, Beijing 100024, China; 3 Research center of Beijing Tourism Development, Beijing 100024, China; 4 School of Business Administration, Southwestern University of Finance and Economics, Chengdu 611130, China; lvshining@126.com; 5 Management School, Tianjin University of Commerce, Tianjin 300134, China; xingbomaggie@126.com

**Keywords:** visitor engagement, environmentally responsible behavior, relationship quality, environmental clue, sustainable development of destinations

## Abstract

Visitor environmentally responsible behavior (ERB) is helpful for promoting the sustainable development of tourist destinations. Existing studies on visitor ERB tend to either focus on restraining visitors’ environmental misconducts or rely on visitors’ psychological factors. Based on the theory of engagement, this paper constructs a theoretical model to explain visitors’ self-conscious ERB. Visitor engagement with the destination is investigated as an independent variable which leads to the improvement of relationship quality and visitor ERB. Relationship quality is explored to mediate the impact of visitor engagement on ERB. In addition, the moderating role of environmental clue on the tested relationships is also examined. This study adopts partial least squares structural equation modeling (PLS-SEM) to investigate a total of 410 valid questionnaires. The results show that (1) visitor engagement with the destination positively affects visitor ERB; (2) relationship quality mediates the impact of visitor engagement on visitor ERB; (3) environmental clue plays a significant moderating role in the effects of engagement and relationship quality on visitor ERB. The study extends theoretical perspectives on visitor ERB and customer engagement, and provides managerially practical value to better understand visitors’ self-conscious ERB.

## 1. Introduction

In the era of mass tourism, the negative environmental effects caused by tourism activities, such as flower and twig picking, lawn trampling [1], animal and plant habitat disturbance, environmental pollution [2], ecological system disturbance [3], the increasing of congestion in destinations [4], and so on, bring tremendous pressure on the management and sustainable development of tourist destinations. Unfortunately, some of the negative environmental impacts result from a range of improper behaviors from visitors. Thus, reducing visitors’ environmentally irresponsible behaviors is crucial to the protection of destinations’ environments so as to achieve the sustainable development of destinations [2,3,4,5]. As a result, visitor environmentally responsible behavior (hereinafter referred to as ERB) has become an important research issue in the field of tourism study in recent years.

Visitor ERB refers to the actions taken by visitors to solve environmental problems and address environmental issues. In order to fulfill their own responsibilities [6], minimize their negative environmental impacts [7], and contribute to the sustainable development of destinations, visitors would like to act and make choices with more considerations on environmental conservation during their tourism activities [8]. Scholars have conducted many studies on the concept of visitor ERB [6], measurement dimensions [8], influencing factors and formation mechanism [9,10], etc. Due to the significance of visitor ERB in the sustainable development of destinations, extant literature on visitor ERB especially focused on the antecedents of visitor ERB and found a lot of influencing factors, which established two main research routes of visitor ERB. 

In one of them, the researchers put emphasis on making use of destinations’ management and environmental protection regulations so as to restrict visitors’ environmental misconducts. Research findings show that persuasive signboards in scenic spots [11], environmental managing policies of scenic spots or destinations [1,10,12], and visitors’ behavior controlling measures [13] all have obvious persuasive and normative effects on visitors’ environmentally improper behaviors. In the other one, the researchers focus on encouraging visitor ERB by the aid of their own psychological factors. The results support the statement that visitors’ personal environmental attitudes [14,15,16], environmental knowledge [5], ethics and personal norms [14,17,18,19,20], sense of responsibility [21,22], and place attachment [23,24,25] can effectively stimulate their ERB. The literature aforementioned made great theoretical and practical contributions to the limitation of visitors’ environmentally unfriendly behaviors and the stimulation of their ERB. However, putting emphasis on destinations’ persuading, warning, restricting, and even punishing means may cause visitor passive emotions and rebellious psychology. Then they may tend to understand the relationship between themselves and destinations as “antagonistic”, psychologically resist the environmental protection regulations from destinations and scenic spots, and continue to deliberately implement environmentally improper behaviors, considering that they can get away with the supervision and punishment from destinations or scenic spots. At the same time, it is difficult to stimulate visitors’ sincere love for the environment of destinations and scenic spots. Hence, the persuading, warning, restricting, and punishing means may cause high supervision cost and non-sustained effects for destinations and scenic spots [26]. In contrast, it may be a better way to center on the use of visitors’ psychological factors to stimulate their ERB. However, in such cases, destinations can only rely on visitors’ personal characteristics instead of other effective measures to stimulate visitor ERB. Additionally, destination managers cannot change visitors’ personal characteristics such as their values, environmental knowledge, and environmental attitudes within the destination. In addition, most scholars assessed the impacts of visitors’ psychological factors on their ERB in usual environment, without particular consideration of tourism’s “unusual environment” [27]. As we all know, the tourism setting is different. Actually, tourism is the overflow from daily life and is composed of different phenomena compared with the usual environment, such as visitor’s shift from work to play, from self-discipline to self-indulgence. Consequently, visitors are easy to take actions from ethics to moral disorders [28], which often make visitors’ psychological factors less predictive for their ERB in a tourism setting. Visitor ERB is the result of the interaction between an individual visitor and tourism setting of a destination [1]. Destination managers need to think about how to effectively guide visitors to love the destination as their own home and proactively take ERB. Thus, what measures destination managers can take to effectively stimulate visitors’ conscious and active ERB is the main object of our present paper.

We know that a marketing-induced idea can change customers’ motivational state and behavioral intention, which provides a new approach to promote visitor ERB from the perspective of marketing. In 2001, Gallup Management Journal put forward the concept of “customer engagement” in an article entitled “The Constant Customer” for the first time [29], which was sought after by marketing business and academia. In 2010, a special issue on the theme of “Customer Engagement” was published in Journal of Service Research [30], which further promoted the academic enthusiasm on customer engagement. Customer engagement has become a hot research topic in the field of marketing in the 21st century. Although the research on customer engagement is still in its infancy, scholars have achieved consensus on that the engagement is a long-term, valuable, and intimate interaction between engagement subject and object [31], as well as reflects the intensity of emotional, psychological, and behavioral connection to the focal object [32,33,34]. Therefore, the construct of engagement is helpful to promote customers’ positive emotional and behavioral inputs to products or brands, which can bring in a series of positive results by influencing customers’ behaviors [35]. A strong body of literature especially emphasizing the positive outcomes of customer behaviors has emerged in the research field of engagement outcomes. For example, some scholars support the idea that customer engagement with a brand can help to promote customers’ trust and emotional commitment to the brand [36,37], higher level of satisfaction and loyalty with the brand [38,39,40], and positive intention of word-of-mouth [41]. Additionally, other scholars believe that as a higher quality, deeper level, and more meaningful subject–object relationship, customer engagement can lead to more consequences beyond the superficial results including satisfaction and loyalty. Engagement can bring in favorable outcomes that go beyond transactional motivation, such as customers’ active participation to virtual community, value co-creation, and knowledge sharing behavior, etc. [33,42,43]. The consequences of customer engagement are expanded from transactional value to non-transactional value. 

However, no evidence shows whether engagement can affect customers’ prosocial behaviors such as ERB. However, some studies point out that customer engagement should be included in relationship marketing, and the core concepts of relationship marketing can be extended to the relevant research of customer engagement [44]. This means that by improving the engagement degree of customers (in the context of tourism settings, customers are visitors), it can help to enhance the relationship quality between visitors and destinations. Some present literature on visitor ERB support that relationship quality can be an important antecedent variable of visitor ERB [45,46]. As a result, we predict and theorize that visitor engagement can help to strengthen the relationships between destinations and visitors, so as to stimulate their ERB in tourism activities. Meanwhile, we predict that even visitors with high engagement or relationship quality with a destination may carry out environmentally irresponsible behaviors because of tourism’s “unusual environment”. Destinations’ appropriate environmental clues may help visitors to adjust their improper environmental behaviors or acquire professional environmental knowledge. Based on this, the current study aims to explore the relationships among the constructs of visitor engagement, relationship quality, environmental clue, and visitor ERB. It is expected that the results of the study will provide managerial implications for destinations to stimulate visitors’ conscious and proactive ERB, and make theoretical contributions to the existing literature on the antecedents of visitor ERB as well as the consequences of customer engagement.

## 2. Theory and Hypothesized Model

### 2.1. Visitor Engagement and Their Environmentally Responsible Behavior

Why are visitors more likely to conduct environmentally irresponsible behaviors in the context of tourism than in their daily lifestyle? The reason may be that they regard the destination as the product of their consumption. Therefore, in their understanding the relationship between themselves and the destination is antagonistic. Visitor engagement refers to visitors’ non-transactional cognitive (e.g., interest in the destination), emotional (e.g., feeling about the destination), and behavioral (e.g., behaviors related to the destination) connection toward a destination [47], which occurs through visitors’ interactive and co-creative experiences with the destination as a whole and its products, services, and activities [48]. The concept of engagement focuses on visitors’ connection, attachment, emotional involvement, and participation with the destination, and represents visitors’ positive psychological and emotional attachment to a destination [32,49]. Thus, engagement can improve the relationship between visitors and the destination environment, as a result, visitors might be more willing to eliminate their antagonistic feeling toward destinations, would be more likely to treat a destination’s environment as their homelike environment, and feel guilty damaging a destination’s environment. Additionally, by promoting a strong connection between visitors and a destination, engagement is helpful to generate visitors’ positive emotions toward the destination. The generation of positive emotions will have a significant impact on visitors’ behavioral intention. Research on influencing sustainable behaviors has begun to examine the role of positive emotion. The findings show that the driving effect of individual’s positive emotions on their green purchase behavioral intention cannot be ignored [50]. Other studies show that the stronger the emotional connection between visitors and national parks, the more willing visitors are to take the initiative to carry out ERB, such as picking up other peoples’ litter, giving up visiting their favorite places for environmental reasons, paying higher entrance fees, and working on park projects [51]. In addition, visitors’ positive emotion, awe towards the environment of a destination, is also positively related to the implementation of their ERB [52]. Based on the engagement theory and previous studies, the current study predicts that engagement can lead to the consequence of visitor ERB by promoting a strong connection between visitors and a destination and making visitors generate positive emotions towards a destination.

**Hypothesis 1** **(H1).** *Visitor engagement is positively related to visitor environmentally responsible behavior.*

### 2.2. Visitor Engagement and the Quality of Relationship between Visitors and Destination

Engagement is an important concept in the field of relationship marketing in the 21st century. It is generated from the continuous and frequent interaction between subject and object [31], and reflects the strength and continuity of the relationship between them. According to Vivek et al., the research on engagement should be included in the research of relationship marketing. Engagement is especially helpful to establish a more in-depth, meaningful, and lasting interaction between the subject and object, so as to bring in positive and valuable results to both the subject and object [44]. The research findings show that engagement can result in positive transactional value, influencing value, knowledge value, and recommendation value to the object [53]; and generate positive functional value, emotional value, and self-realization social value to the subject [54]. For example, enterprises can benefit from increasing brand equity, sales, and profits from truly engaged customers, and customers can be more satisfied with the enterprises through their engagement programs. By creating value for both the subject and the object, engagement could help to improve the quality of relationship between them. Similarly, visitor engagement reflects the strength and development ability of the relationship between visitors and destination. Highly engaged visitors will show positive psychological and behavioral responses to the destination, and for themselves they will obtain the identification of identity, the enhancement of pleasure and happiness, as well as the realization of self-value in the process of engaging with a destination [49]. The combination of positive emotions as well as values brought to visitors is conducive to promoting visitors’ recognition of a destination and higher emotional commitment to a destination, so as to improve the quality of the human–place relationship between them. Extant literature shows that brand engagement is positively related to the quality of the relationship between customers and brands [36,49]. Therefore, it can be predicted that visitor engagement can also have a positive impact on the quality of the relationship between visitors and destination.

**Hypothesis 2** **(H2).** *Visitor engagement positively affects the quality of the relationship between visitors and destination.*

### 2.3. Relationship Quality and Visitor Environmentally Responsible Behavior

The close link between brand relationship quality and customer positive behaviors has been confirmed by a large number of studies [55,56]. Therefore, improving the quality of the relationship has become an effective means to stimulate customers’ positive behaviors. For example, studies show that the quality of the relationship between a hotel and its customers is conducive to promoting the occurrence of positive behaviors of customers, such as share of purchases, relationship continuity, and word-of-mouth [57,58,59]. Visitor ERB is also a kind of positive behavior. The higher the quality of the relationship between visitors and destination, the more likely they will regard the interests of the destination as part of their own interests, and they are more willing to make higher environmental commitments to the destination, so as to take responsible behaviors during their tourism activities in the destination. On the contrary, if visitors’ perception of the relationship quality is poor, they will have negative attitudes towards the destination and increase the negative tendency of behaviors. He et al. confirmed that the better the relationship quality between residents’ perception and destination, the more helpful it is to stimulate resident ERB [45]. A study on visitors also showed that the quality of perceived relationship between visitors and destination has a positive impact on their ERB [46]. Based on the above reasoning and literature, this study proposes the following hypothesis.

**Hypothesis 3** **(H3).** *Visitor environmentally responsible behavior is positively related to the quality of the relationship between visitors and destination.*

### 2.4. Moderating Role of Destinations’ Environmental Clues

Even visitors with high engagement or relationship quality with a destination may carry out environmentally irresponsible behaviors in their tourism activities. In one case, visitors may implement environmentally irresponsible behaviors unconsciously. In this case, visitors know what kind of behaviors are unfriendly, but they may temporarily fail to implement ERB due to the impulsiveness of the tourism experience and relaxed atmosphere of tourism context. For example, in face of the beautiful scenery, visitors may unconsciously ignore the landscape path and walk straight across the lawn to enjoy the beautiful scenery. Therefore, destinations only need to remind visitors appropriately, and visitors’ rationality will urge them to strengthen their code of conduct and implement ERB. In another case, visitors may also carry out environmentally irresponsible behaviors due to their lack of professional environmental knowledge. For instance, visitors may take photos in some cultural relics or touch cultural relic sculptures. In this case, it is possible that visitors do not realize that their behaviors are irresponsible. Appropriate environmental clues in destinations or scenic spots will help visitors acquire professional environmental knowledge which is conducive for visitors to adjust their behaviors. Previous studies have shown that scenic spots’ signboards messages, as a kind of widely used environmental clue in destinations, have an obvious persuasive effect on visitor ERB [11]. Based on this, the study makes deductions that appropriate destination environmental clues can moderate the impacts of visitor engagement and the relationship quality on visitor ERB.

**Hypothesis 4** **(H4).** *Destinations’ environmental clues can moderate the effect of visitor engagement on visitor environmentally responsible behavior.*

**Hypothesis 5** **(H5).** *Destinations’ environmental clues can moderate the effect of relationship quality on visitor environmentally responsible behavior.*

**Figure 1:**
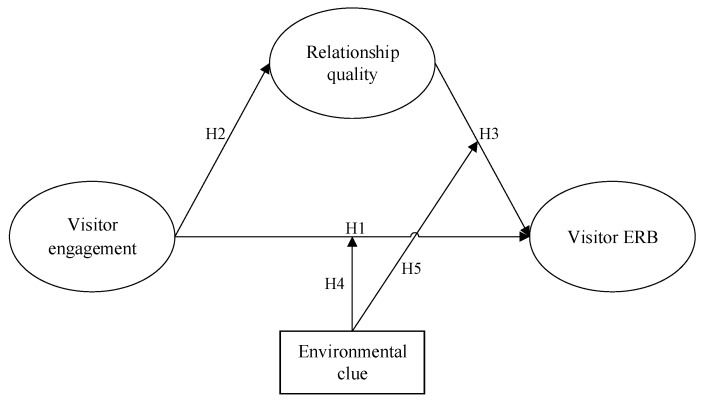
Research model.

Based on the engagement theory and relevant literature, a conceptual framework is proposed (Figure 1).

## 3. Measurement and Data Collection

### 3.1. Measures

In the field of tourism research, the scale of brand engagement developed by So [60] has been widely adopted by scholars [48,49]. However, the scale from So is more suitable to measure visitors’ engagement with micro tourism enterprises’ brands, which is quite different from the measurement of visitor engagement in the current study. The scale developed by Jiseon et al. based on visitors in a comprehensive tourism resort is more similar to the situation in this study, and its measurement of visitor engagement is based on scholars’ conceptualized engagement as a multidimensional construct covering cognitive, emotional, and behavioral dimensions by 10 items. Therefore, in this study visitor engagement was determined by means of 10 items adapted from Jiseon et al. [47].

Relationship quality is also a multidimensional construct with subconstructs such as trust, commitment, satisfaction, and so on. Taking the purpose of this research into consideration, relationship quality was evaluated referring to He’s research [45] by means of four items measuring visitors’ environmental commitment to a destination [61] and one item to measure visitors’ overall satisfaction with the destination.

For visitor ERB, different scholars developed different scales to measure it based on different research purposes. Although some scholars used a single item to measure it, most scholars agreed on using multiple variables to measure. Among them, the measurement scale from Lee et al. has been widely adapted, which consisted of 24 items measuring seven dimensions of ERB [8]. However, considering that the ERB in this study refers to the ERB of visitors during tourism activities in a destination, excluding the general ERB of visitors, this study evaluates visitor ERB by referring to previous studies [1,8,46,62]. At last, eight items were selected after pilot confirmatory factor analysis. Seven-point Likert-type response options ranging from 1 (strongly disagree) to 7 (strongly agree) was utilized for all the measurement scales above.

A destination’s environmental clue refers to whether visitors perceived or noticed the messages about their ERB during their tourism activities. Thus, this study measured it by directly asking visitors whether they perceived or noticed the environmental messages about their behaviors during their tourism activities with 1 representing “Yes” while 0 represented “No”.

### 3.2. Data Collection and Sample

In order to improve the questionnaire’s structure and content validity, the questionnaire was pilot tested first. The researchers first invited some college students to read the measurement items to make sure there were no linguistic or cognitive ambiguities. Then, a preliminary survey was conducted on researchers’ friends who had recently traveled. Of 100 distributed questionnaires, 96 valid ones were retrieved. The reliability and validity of the collective questionnaires were tested, and the results showed that the Cronbach’s alpha of each latent variable was greater than 0.7, and the factor loading of each measurement item was greater than 0.5 at the significant level of 0.01, indicating that the scale had good reliability and validity and could be used for large-scale formal investigation.

Official data collection was conducted from visitors existing in two destinations (Taiyuan, Shanxi province and Jinan, Shandong province) located in central China from September 20th to October 31st in 2018. It is the peak season for China’s tourism, therefore, both destinations attracted a large number of visitors and faced severe environmental problems during that time. Thus, it is important to understand whether visitor engagement can affect their ERB so as to provide effective managerial implications for destinations. Potential respondents were asked if they had fully explored the destination and were encouraged to communicate with the researchers if they were uncertain or confused about what was being asked on the questionnaire. A total of 444 questionnaires were collected. However, after removing the invalid questionnaires, 410 were used in our empirical research, with an effective rate of 92.3%. Among the effective questionnaires, men accounted for 49.0% and females for 51.0%. A total of 85.1% of the respondents were aged between 18 and 40 and they were generally highly educated (66.1% have obtained a bachelor or master degree). A total of 70.7% of the respondents reported an average income of less than 8000 ¥ per month (Table 1).

## 4. Results

### 4.1. Measurement Model

According to Hair et al., partial least squares structural equation modeling (PLS-SEM) is perfectly suitable for predictive research and theoretical development [63], which is similar to that of this study. Additionally, strictly normal distribution of measured variable data is not required in PLS-SEM [64]. Therefore, PLS-SEM was used to test the reliability, validity, and causality of the theoretical model in this study. 

PLS algorithm in Smart PLS 2.0 was used to evaluate the measurement model’s reliability and validity. The results are shown in Table 2. A model is considered reliable when the Cronbach’s alpha and composite reliability of each latent construct is equal to or greater than 0.70 [65]. In this study Cronbach’s alpha and composite reliability range from 0.87 to 0.93, exceeding the threshold value of 0.7, indicating that the reliability of the measurement model is satisfactory. The factor loadings of 10 observed variables for visitor engagement range from 0.51 to 0.83, 0.73 to 0.89 for five items of relationship quality, and 0.57 to 0.87 for eight items of visitor ERB, all exceeding 0.5 [65,66,67] and indicating that the scale has a good convergent validity. Additionally, each variable’s factor loading on its construct is greater than all of its loadings on other constructs (Table 3). Average variance extracted (AVE) value of each latent construct ranges from 0.51 to 0.67, larger than the acceptable requirement of 0.5. According to Table 4, the square root of AVE value of each construct is larger than the correlation coefficients with other constructs, suggesting that the constructs have good discriminant validity. All these show that the variables used to represent the model constructs are satisfactory. 

### 4.2. Structural Model

**Figure 2:**
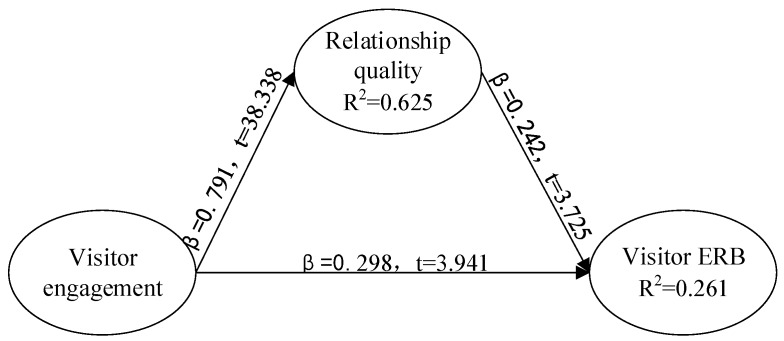
The path analysis results of structural equation modeling (SEM).

Path coefficients can measure the strength of the relationship between latent variables of the model, where the significant value should be at least 0.05 [68]. Smart PLS2.0 was used to test the structural model. The path coefficients and their significant values are listed in Figure 2. It can be seen that visitor engagement significantly affects visitor ERB (beta1 = 0.298, t = 3.941, *p* < 0.01). Visitor engagement significantly affects the quality of the relationship between visitors and destination (beta2 = 0.791, t = 38.338, *p* < 0.01). The quality of the relationship significantly affects visitor ERB (beta3 = 0.242, t = 3.725, *p* < 0.01). Relationship quality partially plays a mediating role between visitor engagement and visitor ERB. Hypotheses 1, 2, and 3 are supported.

The value of R^2^ describes the degree of explained variance of the dependent latent variables and is used to determine the explanatory power of a structural model [65]. As can be seen in Figure 2, relationship quality is one of the dependent latent variables, and its coefficient of determination R^2^ = 0.625, indicating that relationship quality can be explained by visitor engagement by 62.5% variance. The coefficient of determination of visitor ERB intention is 0.261, suggesting that the variance of visitor ERB intention can be explained by 26.1%. According to Hair et al., the value of R^2^ equaling to or greater than 0.20 is substantial [69] and this study reaches the acceptable standard.

Goodness-of-fit (GoF) represents the overall predictive ability of the model. The value of GoF is the square root of multiplying the mean value of AVE by mean value of R^2^ of latent variables. The overall goodness-of-fit of the model in this study is 0.52, higher than the judgment standard of strong goodness-of-fit (GoF_large_ > 0.36) [70], indicating that the model in this study is satisfactory.

In order to evaluate whether exogenous constructs have a substantive impact on endogenous constructs, the effect size of Cohen’s f^2^ value [71] was used and calculated. The Cohen’s f^2^ value of the model in this study is a little weak, which is 0.131. We also assessed the predictive relevance (Q^2^) to assess the predictive capacity of the model. Both of the endogenous constructs’ Q^2^ (0.495 for relationship quality and 0.147 for visitor ERB) are larger than 0 [72], which means the predictive relevance of the model is significant.

### 4.3. Moderating Role of Environmental Clue

In order to test the moderating role of destinations’ environmental clues, the researchers first divided the samples into two groups according to whether visitors noticed the environmental clue or not during their tourism activities in the destination. The results show that 222 respondents noticed the environmental clue while 188 respondents did not. Then PLS-SEM was used to test the two groups respectively. Path coefficients and their significant results are shown in Table 5. We can see that both the path coefficients of visitor engagement on visitor ERB in the two groups are significant. However, the path coefficient (beta_Yes_ = 0.338, t = 5.288) in the group with respondents noticing the environmental clue is significantly higher than the other group (beta_No_ = 0.219, t = 2.638). The path coefficients of visitor engagement on relationship quality are both significant in the two groups without obvious differences (beta_Yes_ = 0.741, t = 30.994; beta_No_ = 0.764, t = 33.811). The two groups’ path coefficients of relationship quality on visitor ERB are both significant. However, the path coefficient (beta_Yes_ = 0.229, t = 3.367) in the group with respondents noticing the environmental clue is also significantly higher than the group with respondents not noticing the environmental clue (beta_No_ = 0.173, t = 2.852). Hypotheses 4 and 5 are supported, which means that environmental clue can moderate the effects of visitor engagement and relationship quality on visitor ERB.

## 5. Discussion

### 5.1. Theoretical Contributions

As ERB has become increasingly important in the sustainable development of tourist destinations, scholars actively focus on minimizing environmental burdens created by visitors. Changing visitors’ behaviors towards being more environmentally responsible has been considered as one of the most effective strategies for overcoming these burdens.

The existing literature on visitor ERB were generally carried out from the perspectives of psychology, sociology, and management, emphasizing the dependence on visitors’ psychological characteristics or restriction of visitors’ improper behaviors, but research is lacking on stimulating visitors’ proactive responsible behaviors from the perspective of the human–place relationship. This study focuses on exploring the active stimulation strategies of visitor ERB. Based on the theory of customer engagement, it provides new explanations for the occurrence of visitor ERB and finds new driving factors in marketing areas for ERB. The research results have made theoretical contributions to the existing literature on ERB by enriching the theoretical perspectives of studies on visitor ERB. At the same time, previous scholars’ studies on the positive relationship between brand engagement and brand relationship quality are mostly based on micro enterprise brands [36,49]. This study supports the positive impact of visitor engagement on the relationship quality between visitors and destination in the context of tourism activities, therefore improves the external validity of micro brand engagement research. The research result of the positive impact of relationship quality on visitor ERB also supports previous scholars’ research conclusions [45,46].

Customer engagement is an important research topic in the field of marketing in the 21st century. After 2010, American Marketing Science Institute (MSI) has repeatedly set “Customer Engagement” as the research priority of marketing [30]. However, most of the existing literature is still focused on the study of micro brand engagement [33,34,36,37]. This paper, together with the previous research on engagement with tourism resorts [47], expands the research scope of engagement by studying visitor engagement with the destination as a whole tourism product. Meanwhile, in the past two decades of research, scholars have mostly supported the consequences of engagement as satisfaction, loyalty, word-of-mouth, customer feedback, and repurchase intention [35,36,37,38,39,40]. This study focuses on the non-transactional value of visitor engagement and finds that engagement has a positive effect on visitor ERB. The research findings make theoretical contributions to the existing literature in the field of customer engagement by enriching the consequences of engagement and expanding the impact value of customer engagement.

### 5.2. Practical Implications

The findings of this research have several implications for destination managers seeking to understand how they might stimulate visitors to conduct in environmentally responsible ways during their tourism activities.

Destination managers should attach much importance to using engagement to stimulate visitors’ proactive ERB. Through engaging with visitors, destination managers might help to improve the quality of the relationship between visitors and the destination. The new friendship-type human–place relationship might replace the original trading-type antagonistic relationship between them. The improvement of the relationship quality may further stimulate visitors to engage in ERB. Therefore, the destination managers should change their managerial thinking by trying to replace the passive restricting and supervising visitors’ misconducts with inspiring visitors’ proactive ERB by engagement.

Destination managers should provide as many chances as they can to interact and engage with visitors. Engagement is achieved through continuous and interactive experiences with a focal object [35,36,37]. Tourism brand engagement can be strengthened by setting participatory activities for tourists [49]. Therefore, the degree of engagement between visitors and a destination could be gradually enhanced during the interaction between the two. Destination managers should take the initiative to provide visitors with more participation means and platforms, and build special teams to manage the participation activities and platforms of visitors. For example, visitors’ sight, hearing, smell, taste, and touch experiences in their tourism activities is helpful to stimulate visitors to integrate into more emotions and participation, and improve both visitor’s perception of the human–place relationship and the level of visitor engagement. Additionally, the development of Internet and new media has provided more possibilities for destinations to engage with visitors. Destination managers can take the initiative to attract visitors to participate in discussion topics on destinations through their official micro-blog, WeChat, and other media platforms. Finally, the generation of visitor’s emotions, psychological connection, and engagement will be achieved.

Destination managers should make use of the environmental clue to stimulate visitor ERB. On the one hand, destination managers should actively spread environmental knowledge and skill related to visitor ERB during their tourism activities. On the other hand, destination managers should strengthen the guidance and promotion of visitor behavior norms by environmental clue. For example, at the entrance of the scenic spot, visitors should be provided with free interesting environmental knowledge manuals. Warning signboards should be set up in prominent positions in scenic spots to remind visitors of their codes of conduct. Additionally, tour guides and other service personnel should be trained to provide environmental information and knowledge to visitors during their serving process.

### 5.3. Limitations and Future Research

Identifying the antecedents of visitor ERB is helpful to provide implications for destination managers’ decision-making for sustainable tourism development. This paper tried to explore the predicting factors of visitor ERB from the perspective of marketing and empirically tested the impact mechanism of visitor engagement on their ERB. However, limitations of our paper are worth discussing. 

Firstly, the construct of visitor engagement focuses on visitors’ connection, attachment, and emotional involvement with a destination [48,49]. It represents visitors’ sincere love for a destination. However, just as we say that people fall in love with a place because they love the people there, the same is true for visitors, which means that visitors may love a destination because they love the residents there. Therefore, residents may play a major role in improving visitor engagement with a destination. Especially, with the popularity of P2P accommodation, the interaction between visitors and local residents is no longer brief, arguable, and superficial. Visitors may experience affective bonds and positive sentiments with local residents, which is referred to as “emotional solidarity” by scholars [73,74]. Implicit in the construct of solidarity is its potential link to visitor engagement with a destination. To date, however, in tourism studies, emotional solidarity with residents of the destination has not been used to explain visitor engagement. Therefore, it is necessary to promote the research in this field by applying the theory of emotional solidarity to examine the relationships of visitors’ emotional solidarity predicting visitor engagement and then visitor ERB. Taking measures to promoting visitor ERB is extremely important to the future of sustainable tourism development of destinations. The work surrounding visitor ERB still needs more extensive discussion and quite varied research perspectives in future. 

Secondly, only visitor engagement was considered as a predictor variable within our research model and the total variance of visitor ERB explained is only 26.1%. As such, there is a strong need for understanding the psychological processes that underpin visitor ERB. Therefore, a host of other psychological constructs that may serve to explain more variance in the construct need to be added to the model. Discussion on visitor ERB from an integrated model including variables from both marketing and psychology needs to be done in future study, which can account for greater variance in visitors’ intent to engage in environmentally responsible behavior.

Lastly, similar to most of the research on ERB in the tourism context, we primarily measured visitor ERB which refers to the behaviors mitigating their direct environmental impact by a single-factor scale with six items. However, as Landon et al. [20] suggested, the scope of behaviors relevant to sustainable tourism is much larger and we need to measure visitor ERB across the triple bottom line (including “Willingness to Sacrifice”, “Localism”, and “Eco-behavior” constructs). Therefore, there is a strong need for redefining and remeasuring visitor ERB in future and for empirically testing the impacts of visitor engagement on the three types of ERB constructs (the work of Landon et al. [20] serves as an example).

## 6. Conclusions

With the rapid development of tourism, destinations are faced with severe environmental problems due to the conscious or unconscious environmental misconducts of visitors. Encouraging visitors to adopt environmentally friendly behaviors will help to minimize environmental burdens created by them. Therefore, it is very important to identify the key antecedents and influence mechanism of visitor ERB. Based on the theory of customer engagement and previous literature, the current study established and assessed a model to explore the active driving mechanism of visitor ERB, and the main findings are as follows:

(1) Visitor engagement with destinations and scenic spots can positively affect visitor ERB during their tourism activities. Visitor engagement is characterized by repeated interactions between visitors and a destination so as to strengthen the psychological, emotional, and behavioral inputs from visitors [36]. Additionally, they may also invest more enthusiasm and attention in engaging with a destination [32,35]. In this process, visitors could regard the destination as their friend rather than a product. Therefore, they would not damage the destination including its environment deliberately, and they are more willing to take environmental responsibilities in their tourism activities.

(2) Relationship quality partially mediates the effect of visitor engagement on their ERB. Engagement is a new concept in marketing areas in the 21st century and represents a positive psychological and emotional state between subject and object. Engagement with tourism brands can improve the relationship quality between tourists and hotels or airline companies [49]. Previous literature also indicated that relationship quality can play a significant role in predicting visitor ERB [45,46]. We support their opinions by testing the model in this study. Engagement is a relationship-based construct and is conducive to improving the quality of the relationship between visitors and destination, so as to promote visitors to engage in a series of positive behaviors, including ERB. In addition, traditionally, because of visitors’ curiosities, they would prefer to visit a new destination rather than revisit a destination second time. Therefore, destinations have difficulties in establishing a long-term friendly relationship with visitors. The current study shows that engagement can become an important means to solve this problem.

(3) A destination’s environmental clue can strengthen the impacts of visitor engagement and relationship quality on their ERB. The effects of visitor engagement and relationship quality on ERB are significantly higher when respondents noticed destination’s environmental clue than when they did not. Environmental clue plays a positive moderating role in the impacts of visitor engagement and relationship quality on visitor ERB. A destination’s relaxing atmosphere and the characteristics of tourism activities such as temporary and unusual environment, tend to weaken visitors’ behavioral norms. At some specific destinations, visitors sometimes cannot realize their improper conducts. A destination’s appropriate environmental clue can help to transfer visitor engagement into actual ERB.

## Figures and Tables

**Table 1:** Sample characteristics.

Variable	*n*	%	Variable	*n*	%
**Gender**			**Monthly Income**		
Male	201	49.0	Less than 5000RMB	190	46.3
Female	209	51.0	5001–8000	100	24.4
**Age (years)**			8001–17,000	88	21.5
18–30	190	46.3	17,001–40,000	28	6.8
31–40	159	38.8	More than 40,000	4	1.0
41–50	37	9.0	**Level of Education**		
51 or Older	24	5.9	Less than High School	4	1.0
			High school/Technical school	135	32.9
			Undergraduate/Associate Degree	260	63.4
			Postgraduate Degree	11	2.7

**Table 2:** Reliability and convergent validity of the questionnaire.

Constructs and Scale Items	Factor Loadings	Cronbach’s Alpha	Composite Reliability	AVE
**1. Visitor Engagement**		0.89	0.91	0.51
I think about the destination while visiting it	0.54			
I would like to learn more about the destination	0.51			
I think about the destination even after visiting it	0.67			
I feel very positive when I visit the destination	0.79			
Visiting the destination makes me happy	0.80			
I feel great when I visit the destination	0.83			
I am very proud to visit the destination	0.76			
I would like to spend more time in the destination	0.78			
I would like to visit the destination frequently	0.82			
I would like to pay more attention to the destination	0.66			
**2. Relationship Quality**		0.87	0.91	0.67
I am committed to keeping the best interests of the environment in mind at the destination	0.73			
I am interested in strengthening my connection to the environment of the destination in future	0.89			
I feel very attached to the environment of the destination	0.82			
I expect that I will always feel a strong connection with the environment of the destination	0.85			
I am satisfied with my visit to the destination	0.79			
**3. Visitor Environmentally Responsible Behavior**		0.91	0.93	0.63
I would like to follow the legal policies of the destination and scenic spot	0.80			
I would like to dispose of the garbage properly during my trip	0.84			
I would like to protect the plants and animals of the destination and scenic spot	0.87			
I would like to protect the relics and facilities of the destination and scenic spot	0.87			
I would like to encourage others to follow the legal policies of the destination and scenic spot	0.85			
I would like to encourage others to protect the environment of the destination and scenic spot	0.80			
When I see garbage from others, I will pick them up and put them in the trash	0.57			
I try to stop others from damaging the environment of the destination and scenic spot	0.67			

**Table 3:** Crossing loadings of items on constructs.

Scale Items	Visitor Engagement	Relationship Quality	Visitor ERB
I think about the destination while visiting it	0.54	0.38	0.29
I would like to learn more about the destination	0.51	0.35	0.38
I think about the destination even after visiting it	0.67	0.48	0.36
I feel very positive when I visit the destination	0.79	0.57	0.41
Visiting the destination makes me happy	0.80	0.59	0.44
I feel great when I visit the destination	0.83	0.61	0.39
I am very proud to visit the destination	0.76	0.63	0.30
I would like to spend more time in the destination	0.78	0.64	0.33
I would like to visit the destination frequently	0.82	0.63	0.29
I would like to pay more attention to the destination	0.66	0.59	0.26
I am committed to keeping the best interests of the environment in mind at the destination	0.55	0.73	0.42
I am interested in strengthening my connection to the environment of the destination in future	0.67	0.89	0.35
I feel very attached to the environment of the destination	0.58	0.82	0.35
I expect that I will always feel a strong connection with the environment of the destination	0.62	0.85	0.29
I am satisfied with my visit to the destination	0.65	0.79	0.48
I would like to follow the legal policies of the destination and scenic spot	0.37	0.36	0.80
I would like to dispose of the garbage properly during my trip	0.38	0.36	0.84
I would like to protect the plants and animals of the destination and scenic spot	0.39	0.35	0.87
I would like to protect the relics and facilities of the destination and scenic spot	0.37	0.34	0.87
I would like to encourage others to follow the legal policies of the destination and scenic spot	0.37	0.37	0.85
I would like to encourage others to protect the environment of the destination and scenic spot	0.33	0.33	0.80
When I see garbage from others, I will pick them up and put them in the trash	0.41	0.41	0.57
I try to stop others from damaging the environment of the destination and scenic spot	0.38	0.42	0.67

**Table 4:** Discriminant validity of constructs.

Constructs	1	2	3
1. Visitor Engagement	**[0.71]**		
2. Relationship Quality	0.70	**[0.82]**	
3. Visitor Environmentally Responsible Behavior	0.49	0.48	**[0.79]**

Note: [ ] represents the square root of AVE of each latent construct.

**Table 5:** Grouping SEM analysis results.

Predicted Relationships	Standard Path Coefficient		T Value	
Environmental Clue	Yes	No	Yes	No
Visitor engagement → Environmentally responsible behavior	0.338	0.219	5.288	2.638
Visitor engagement → Relationship quality	0.741	0.764	30.994	33.811
Relationship quality → Environmentally responsible behavior	0.229	0.173	3.367	2.852
